# Synergistic Interactions of Eugenol-tosylate and Its Congeners with Fluconazole against *Candida albicans*

**DOI:** 10.1371/journal.pone.0145053

**Published:** 2015-12-22

**Authors:** Aijaz Ahmad, Mohmmad Younus Wani, Amber Khan, Nikhat Manzoor, Julitha Molepo

**Affiliations:** 1 Department of Oral Biological Sciences, School of Oral Health Sciences, Faculty of Health Sciences, University of the Witwatersrand, 7 York Road, Parktown, Johannesburg, South Africa; 2 Departmento de Quimica, FCTUC, Universidade de Coimbra, Rua Larga, Coimbra, Portugal; 3 Department of Internal Medicine, Faculty of Health Sciences, University of the Witwatersrand, 7 York Road, Parktown, Johannesburg, South Africa; 4 College of Applied Medical Sciences, Taibah University, Al-Madinah Al-Munawarah, KSA; 5 Department of Biosciences, Jamia Millia Islamia, New Delhi, Delhi, India; National University of Singapore, SINGAPORE

## Abstract

We previously reported the antifungal properties of a monoterpene phenol “Eugenol” against different *Candida* strains and have observed that the addition of methyl group to eugenol drastically increased its antimicrobial potency. Based on the results and the importance of medicinal synthetic chemistry, we synthesized eugenol-tosylate and its congeners (E1-E6) and tested their antifungal activity against different clinical fluconazole (FLC)- susceptible and FLC- resistant *C*. *albicans* isolates alone and in combination with FLC by determining fractional inhibitory concentration indices (FICIs) and isobolograms calculated from microdilution assays. Minimum inhibitory concentration (MIC) results confirmed that all the tested *C*. *albicans* strains were variably susceptible to the semi-synthetic derivatives E1-E6, with MIC values ranging from 1–62 μg/ml. The test compounds in combination with FLC exhibited either synergy (36%), additive (41%) or indifferent (23%) interactions, however, no antagonistic interactions were observed. The MICs of FLC decreased 2–9 fold when used in combination with the test compounds. Like their precursor eugenol, all the derivatives showed significant impairment of ergosterol biosynthesis in all *C*. *albicans* strains coupled with down regulation of the important ergosterol biosynthesis pathway gene-*ERG11*. The results were further validated by docking studies, which revealed that the inhibitors snugly fitting the active site of the target enzyme, mimicking fluconazole, may well explain their excellent inhibitory activity. Our results suggest that these compounds have a great potential as antifungals, which can be used as chemosensitizing agents with the known antifungal drugs.

## Introduction

Natural products have been regarded as rich sources of potential chemotherapeutic agents. However, due to high minimum inhibitory concentrations, most of them are often overlooked, which results in a decline of investment in this area, despite the fact that natural products are a source of new drugs [1]. In view of this, medicinal chemists started modifying the natural products to synthesize modified analogs and derivatives with improved potency and safety [2]. The modified analogues, sometimes referred to as semi-synthetic drugs, are of great scientific focus and a real advancement is the use of drug combinations, with known mode of action for the treatment of life threatening diseases. Drug combinations have already been in use for the treatment of diseases like cancer, HIV or cardiovascular diseases and it is believed that drug combinations are better at controlling complex diseases with minimal resistance [2,3]. Although the potential of combining natural products or their semi-synthetic analogues with synthetic drugs or introducing these into conventional treatment regimes are not yet systematically explored, the field is abound with opportunities [2].

In antifungal chemotherapy the heterocyclic compounds “azoles” have been immensely researched and currently these are the most popular class of antifungals used in medicine. These compounds bind as the sixth ligand to the heme group in CYP51, changing the structure of the active site and acting as non-competitive inhibitors [4]. Several studies are devoted to validate the potency of azoles as inhibitors of 14 α-demethylase [5]. Impairment of ergosterol biosynthesis by these compounds has also been documented [4,6]. However, prolonged use of some azoles as antifungal agents has resulted in the emergence of drug resistance among certain fungal strains [7–9]. Genetic mutations have been found to be possibly responsible for the resistance to clinically used drugs especially fluconazole [10]. In addition, resistance to new structurally related azoles such as voriconazole [11], and ravuconazole [12] has also been observed. Consequently, the focus of azole research is beginning to shift towards identifying new efficient drug molecules to circumvent this major obstacle. A novel strategy of generating an effective treatment option is the combined use of the semi-synthetic analogues, with enhanced potency, compared to the precursor natural product and an already established drug [13,14].

Our previous findings on the bioactivity of eugenol revealed that eugenol exerts its antifungal activity by targeting ergosterol biosynthesis pathway [15] and has the potential to convert the fungistatic FLC into a fungicidal drug when used in combination with known antibiotics [16]. Here we chose to synthesize eugenol-tosylate and its congeners and investigated their mechanism of binding to Cytochrome P450 14α-sterol demethylase (CYP51), which are essential enzymes in sterol biosynthesis in fungi. We further determined the antifungal efficacies of these newly semi-synthetic derivatives E1-E6 against different FLC-susceptible and FLC-resistant *Candida albicans* species alone and in combination with fluconazole. The mechanism of action was elucidated by quantifying the ergosterol content in different *Candida* isolates following treatment with semi-synthetic eugenol derivatives and the results were validated by docking studies. The differences in the ergosterol biosynthesis are attributed to the differences in the gene expression patterns and in view of this; real time-PCR (qRT-PCR) of one of the striking gene of ergosterol biosynthesis (*ERG11*) was also included in the study.

## Materials and Methods

Solvents and organic reagents were purchased from Sigma Aldrich and Merck (Germany) and were used without further purification. Elemental analyses were performed on HeraeusVario EL III analyzer. The results were within ± 0.4% of the theoretical values. FT-IR spectra were recorded using a Thermo Nicolet 380 instrument equipped with a Smart Orbit ATR attachment. ^1^H and ^13^C NMR spectra were recorded at ambient temperature on a Bruker AVANCE 400 NMR spectrometer using standard parameters. All chemical shifts are reported in δ units with reference to the residual peaks of CDCl_3_ (δ 7.24, ^1^H NMR; δ 77.0, ^13^C NMR) or DMSO-d_6_ (δ 2.50, ^1^H NMR; δ 39.52, ^13^C NMR). ESI-MS was recorded on a MICROMASS QUATTRO II triple quadrupole mass spectrometer. Precoated aluminum sheets (silica gel 60 F_254_, Merck Germany) were used for thin-layer chromatography (TLC) and spots were visualized under UV light.

### Synthesis of Eugenol-tosylate and its congeners

**Fig 1:**
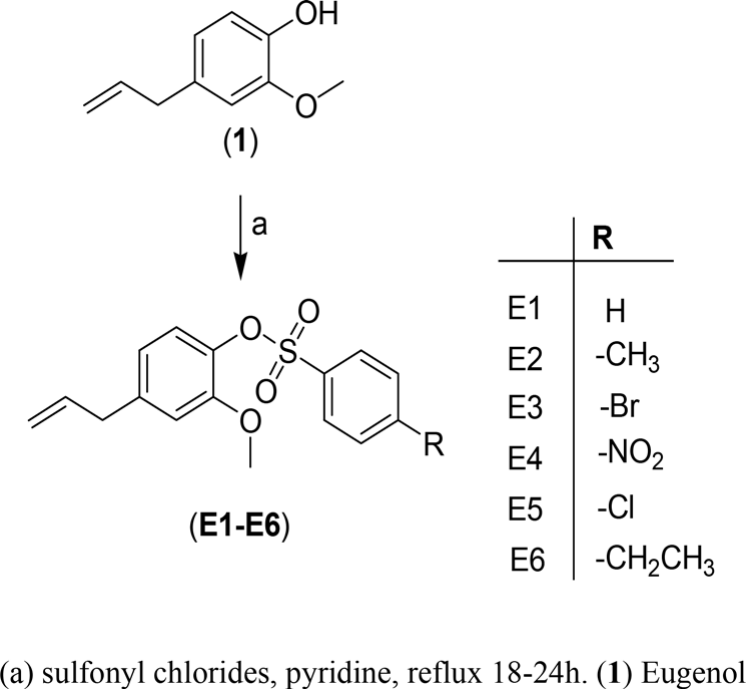
Synthesis of eugenol-tosylate and its congeners E1-E6. (a) Sulfonyl chlorides, pyridine, reflux 18-24h. (**1**) Eugenol

All the compounds E1-E6 were synthesized from 2-methoxy-4-(prop-2-en-1-yl)phenol (1) by treating with different phenyl and substituted phenylsulfonyl chlorides in refluxing pyridine for 18–24 h (completion of reaction was monitored by TLC) (Fig 1). After the completion of reaction the reaction mass was quenched with distilled water and extracted with dichloromethane. Finally the combined organic layer was washed with distilled water again and dried over anhydrous Na_2_SO_4_. All the compounds were obtained as pure oil after removal of the solvent in vacuum.

#### 2-methoxy-4-(prop-2-en-1-yl)phenyl benzenesulfonate (E1)

Yield: 84%; Anal. Calc. for C_16_H_16_O_4_S; C, 63.14; H, 5.30%, found; C, 63.30; H, 5.18%; IR _max_cm^-1^: 3025 (C-H stretch), 1595 (C = C, Ar), 1308, 1150 (S = O); ^1^H NMR (DMSO-*d*_*6*_) (ppm): 7.95–7.50 (4H, m, Ar-H), 7.35 (1H, t, J = 7.8 Hz), 6.84–6.65 (3H, m, Ar-H), 6.32 (1H, m), 4.85 (2H, m), 3.75 (3H, s, OCH_3_), 3.15 (2H, d, J = 15.2 Hz); ^13^CNMR (DMSO-*d*_*6*_) (ppm): 149.8, 143.5, 138.6, 136.2, 134.6, 132.2, 130.1, 129.6, 128.7, 122.8, 118.9, 115.3, 58.6, 45.6; ESI-MS m/z [M+H]^+^ 305.10.

#### 2-methoxy-4-(prop-2-en-1-yl)phenyl 4-methylbenzenesulfonate (E2)

Yield: 85%; Anal. Calc. for C_17_H_18_O_4_S; C, 64.13; H, 5.70%, found; C, 64.28; H, 5.56%; IR _max_cm^-1^: 3033 (C-H stretch), 1587 (C = C, Ar), 1315, 1158 (S = O); ^1^H NMR (DMSO-*d*_*6*_) (ppm): 7.87–7.59 (4H, m, Ar-H), 6.68–6.52 (3H, m, Ar-H), 6.35 (1H, m), 4.78 (2H, m), 3.82 (3H, s, OCH_3_), 3.10 (2H, d, J = 12.5 Hz), 2.25 (3H, s, CH_3_); ^13^CNMR (DMSO-*d*_*6*_) (ppm): 148.9, 144.6, 138.5, 137.0, 135.4, 133.5, 130.6, 129.3, 128.0, 122.5, 118.5, 117.0, 115.7, 56.4, 47.4, 24.8; ESI-MS m/z [M+H]^+^ 319.10; [M+Na]^+^ 342.06.

#### 2-methoxy-4-(prop-2-en-1-yl)phenyl 4-bromobenzenesulfonate (E3)

Yield: 83%; Anal. Calc. for C_16_H_15_O_4_BrS; C, 50.14; H, 3.94%, found; C, 50.26; H, 3.87%; IR _max_cm^-1^: 3020 (C-H stretch), 1572 (C = C, Ar), 1315, 1158 (S = O stretch); ^1^H NMR (DMSO-*d*_*6*_) (ppm): 7.89–7.71 (4H, m, Ar-H), 6.55–6.42 (3H, m, Ar-H), 6.33 (1H, m), 4.96 (2H, m), 4.50 (2H, t, J = 7.5 Hz), 3.98 (2H, t, J = 8.2 Hz), 3.72 (3H, s, OCH_3_), 3.22 (2H, d, J = 14.8 Hz); ^13^CNMR (DMSO-*d*_*6*_) (ppm): 154.2, 149.2, 145.7, 143.6, 136.8, 129.0, 122.4, 118.0, 116.5, 115.4, 56.4, 48.4; ESI-MS m/z [M+H]^+^ 382.98, [M+Na^+^+H]^+^ 406.10.

#### 2-methoxy-4-(prop-2-en-1-yl)phenyl 4-nitrobenzenesulfonate (E4)

Yield: 85%; Anal. Calc. for C_16_H_15_NO_6_S; C, 55.01; H, 4.33, N, 4.01%, found; C, 55.12; H, 4.26, N, 4.15%; IR _max_cm^-1^: 3025 (C-H stretch), 1585 (C = C, Ar), 1380 (NO_2_ stretch), 1310, 1148 (S = O stretch); ^1^H NMR (DMSO-*d*_*6*_) (ppm): 8.45–8.15 (4H, m, Ar-H), 6.85–6.72 (3H, m, Ar-H), 6.30 (1H, m), 4.93 (2H, m), 3.75 (3H, s, OCH_3_), 3.18 (2H, d, J = 15.5 Hz); ^13^CNMR (DMSO-*d*_*6*_) (ppm): 154.2, 149.2, 145.7, 143.6, 136.8, 129.0, 122.4, 118.0, 116.5, 115.4, 56.4, 48.4; ESI-MS m/z [M+H]^+^ 350.06; [M+Na]^+^ 373.10; [M+K^+^+H]^+^ 390.14.

#### 2-methoxy-4-(prop-2-en-1-yl)phenyl 4-chlorobenzenesulfonate (E5)

Yield: 80%; Anal. Calc. for C_16_H_15_O_4_ClS; C, 56.72; H, 4.46%, found; C, 56.84; H, 4.38%; IR _max_cm^-1^: 3033 (C-H stretch), 1587 (C = C, Ar), 1308, 1152 (S = O), 730 (C-Cl); ^1^H NMR (DMSO-*d*_*6*_) (ppm): 7.85–7.62 (4H, m, Ar-H), 6.75–6.55 (3H, m, Ar-H), 6.22 (1H, m), 4.83 (2H, m), 3.85 (3H, s, OCH_3_), 3.18 (2H, d, J = 12.5 Hz); ^13^CNMR (DMSO-*d*_*6*_) (ppm): 148.5, 145.2, 138.2, 136.8, 135.6, 133.5, 132.1, 129.0, 128.1, 122.4, 118.0, 116.5, 115.4, 58.5, 48.2; ESI-MS m/z [M+H]^+^ 339.07; [M+Na^+^+H]^+^ 363.04.

#### 2-methoxy-4-(prop-2-en-1-yl)phenyl 4-ethylbenzenesulfonate (E6)

Yield: 80%; Anal. Calc. for C_18_H_20_O_4_S; C, 65.04; H, 6.06%, found; C, 65.28; H, 6.02%; IR _max_cm^-1^: 3028 (C-H stretch), 1575 (C = C, Ar), 1318, 1143 (S = O stretch); ^1^H NMR (DMSO-*d*_*6*_) (ppm): 7.89–7.71 (4H, m, Ar-H), 6.55–6.42 (3H, m, Ar-H), 6.33 (1H, m), 4.96 (2H, m), 3.72 (3H, s, OCH_3_), 3.22 (2H, d, J = 17.5 Hz), 2.58 (2H, m), 1.29 (3H, s); ^13^CNMR (DMSO-*d*_*6*_) (ppm): 149.8, 144.5, 143.2, 136.1, 128.4, 122.1, 117.5, 115.4, 113.8, 56.3, 48.0, 32.6, 14.8; ESI-MS m/z [M+H]^+^ 333.11; [M+Na]^+^ 356. 08.

### Strains and media

Besides one standard laboratory strain *C*. *albicans* ATCC90028 used as a quality control, fifteen clinical isolates (eight from South Africa and seven from India) of FLC- susceptible and nine FLC- resistant *C*. *albicans* were used in this study (Table 1). Clinical strains were isolated from HIV positive patients and other immunocompromised conditions from the Charlotte Maxeke Johannesburg Academic Hospital, Johannesburg, South Africa and from the Department of Microbiology, All India Institute of Medical Sciences, New Delhi, India. All strains were maintained on YPD (yeast extract, peptone dextrose agar plates). Prior to experiments, growth was initiated by growing one loopful of cells in YPD media up to mid-log phase. All the media and other analytical grade chemicals were purchased from Oxoid, England and E. Merck, India.

**Table 1:** Minimum inhibitory concentrations (μg/ml) of E1-E6 and fluconazole (FLC) against different *C*. *albicans* strains.

Strains		EUG	E1	E2	E3	E4	E5	E6	FLC
**Lab strain**	ATCC 90028	500	16	16	4	8	2	31	4
**Clinical FLC susceptible strains**	074	500	31	31	4	16	2	62	8
	072gr	500	31	31	4	16	2	62	16
	072 big gr	500	31	62	4	31	4	62	16
	079 small gr	500	31	31	4	31	4	31	16
	002 B1	500	8	16	2	2	1	31	8
	003 gr	500	16	32	4	16	4	62	16
	004 gr	500	31	62	8	16	4	62	16
	004 B1	500	8	31	1	4	1	62	8
	177	500	4	16	4	8	2	31	8
	2281	500	31	31	8	16	8	62	8
	2323	500	16	31	8	8	4	31	8
	2367	500	8	16	4	4	2	31	8
	2427	500	8	8	2	4	1	16	8
	2434	500	31	62	16	31	4	62	8
	2508	500	8	31	2	4	1	62	8
**Clinical FLC resistant strains**	3001	500	31	62	16	16	8	62	125
	3048	500	31	62	16	16	8	62	125
	3068	500	16	31	4	8	4	62	125
	3087	500	16	62	8	8	8	62	125
	3081	500	16	16	4	8	2	31	125
	3140	500	31	62	16	16	8	62	125
	2357	500	31	31	8	16	8	62	125
	2261	500	16	31	4	8	2	31	125
	3178	500	8	31	4	8	2	62	125

### Minimum Inhibitory Concentration (MIC)

Two-fold broth microbroth dilution method, as described by the CLSI, was used to determine the MICs of all the newly synthesized derivatives E1-E6 and FLC against different FLC- susceptible and FLC- resistant isolates [17]. All the compounds were diluted using 1% DMSO to yield a concentration of 1000 μg/ml. The positive control fluconazole (1000 μg/ml) and the negative vehicle control (1% DMSO) were also included in every set of experiments. Culture and media controls were also included in every set of experiments to confirm the viability and sterility, respectively. Results were calculated as a mean of the two separate experiments done in triplicate.

### Assessment of the Fractional Inhibitory Concentration Index (FICI)

To determine the interaction of compounds E1-E6 with FLC, microdilution assays were performed as previously described [18]. Briefly, E1, E2, E3, E4, E5, E6 and FLC were added in 1:1 volumes together with 100 μl of media and were serially diluted. Interactions were assessed on the basis of zero-interaction theory of Loewe additivity and FICI values were calculated as follows:

$$FICI=FICa+FICb=\frac{MICa\;in\;combination}{MICa\;tested\;alone}+\frac{MICb\;in\;combination}{MICb\;tested\;alone}$$

Where, MICa is the MIC of the derivatives E1-E6 and MICb is the MIC of FLC. An FICI value of ≤0.5 was interpreted as synergy whereas the FICI values between 0.5 and 1.0 were interpreted as additive. FICI values >4.0 were considered as antagonism and FICI values between 1.0 and 4.0 were considered as indifferent, following the conservative approach as described by van Vuuren [19].

### Varied ratio combinations and isobolograms

Based on the synergistic interactions in 1:1 ratios between E1, E2, E3, E4, E5, E6 and FLC, isobolograms were constructed, where nine different ratios of E1-E6 and FLC were mixed in 9:1; 8:2; 7:3; 6:4; 5:5; 4:6; 3:7; 2:8; and 1:9 ratios, respectively, and their MIC values were then determined. MIC values were also determined for the compounds E1-E6 and FLC, independently. To represent the mean MIC values of these combinations isobolograms were plotted using GraphPad Prism version 5-software as described previously [18]. On examining the data points for each ratio in relation to the MIC values independently, isobolograms were interpreted as synergistic or additive.

### Ergosterol Biosynthesis Assay

Total intracellular sterols from the different *C*. *albicans* cells which were treated with MIC and sub-MIC concentrations of E1 to E6 were extracted and quantified as reported previously using spectrophotometric method (Beckman Coulter) [20]. In every set of experiments untreated (negative control) and fluconazole treated (positive control) cells were also tested. The characteristic four-peaked curve confirmed the presence of ergosterol and 24(28) dehydroergosterol (DHE) in the samples while the flat line depicted the absence of ergosterol in the extracted samples. Ergosterol content was calculated as a percentage of the wet weight of the cell by the following equations:

$$\begin{array}{l} \%\;Ergosterol\;+\%\;24\left(28\right)\;DHE\;=\;\left[\left(A_{281.5}/290\right)\times F\right]/pellet\;weight\\ \%\;24\left(28\right)\;DHE=\left[\left(A_{230}/518\right)\times F\right]/pellet\;weight\\ \%\;Ergosterol\;=\left[\%\;Ergosterol+\%\;24\left(28\right)\;DHE\right]-\%\;24\left(28\right)\;DHE \end{array}$$

Where F is the factor for dilution in ethanol and 290 and 518 are the E values (in percentages per centimeter) determined for crystalline ergosterol and 24(28) DHE, respectively.

### Docking

#### Homology modeling

The 3D model structure of cytochrome P450 lanosterol 14α-demethylase of *C*. *albicans* was built using homology modeling. A flow chart diagram for homology modelling is illustrated in S1 Fig. The amino acid sequence of the enzyme was obtained from the Universal Protein Resource (http://www.uniprot.org) (Accession Code: P10614) and sequence homologous was obtained from Protein Data Bank (PDB) using Blast search. Based on the result of blast search, we found lanosterol of yeast bearing PDB ID: 4K0F with highest homology of 65%. This was selected as a template for modelling our protein (P10614). P10614 was submitted to I-TASSER [21], an online server for modelling and the best model was selected on the basis of higher C-score and least Z score. In parallel we also modelled it manually by modeler [22]. We selected the model with least DOPE score and then we compared the two results with our template. The model with least RMSD was selected as the best one and further used for docking studies. To have the heme group, necessary for the ligand molecules to bind, in our modeled protein, we took the homology modeled structure and aligned it to the templates (PDB code: 3LD6; 4LXJ), containing the heme in its active site using PyMol, then we figured out the atoms that are interacting with it. Finally we incorporated the co-ordinates of heme in our model and also wrote down the interactions as extracted from the template file.

**Fig 2:**
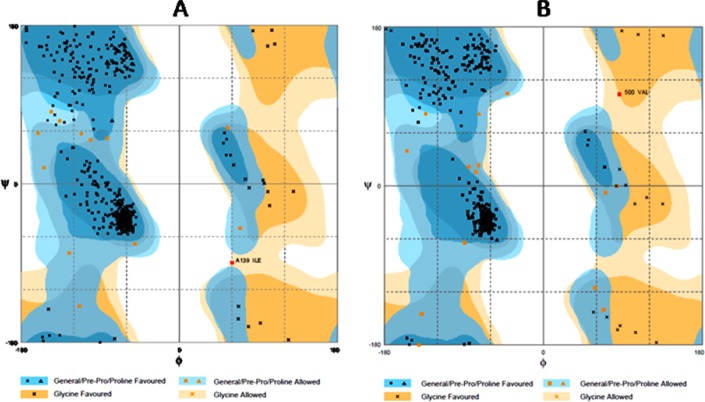
Geometric evaluation of the modeled protein and the template shown as Ramachandran plots for 4KOF (A) and modeled protein (B); 97% of residues were in the favor, 2.9% allowed regions, and 0.2% was in the outlier region as compared to 4KOF template (97.5%, 2.3%, and 0.2%).

Geometric evaluations of the modeled 3D structure were performed using Rampage as shown in Fig 2A and 2B. The Ramachandran plot of our model showed that 97% of residues were in the favor, 2.9% allowed regions, and 0.2% was in the outlier region as compared to 4K0F template (97.5%, 2.3%, and 0.2%). The plot statistics are presented in Table 2. (Also see S2 and S3 Figs).

**Table 2:** Ramachandran plot statistics for the modeled protein and the template^#^.

Structure	Number of residues in favored region (%)	Number of residues in allowed region (%)	Number of residues in outlier region (%)
**4KOF**	97.5	2.3	0.2
**MODEL BUILT**	97	2.9	0.2

^#^Done using RAMPAGE evaluation server

Validation was carried out using ProSA to obtain the Z-score value for the comparison of compatibility (See S4 Fig). The Z-score plot showed spots of Z score values of proteins determined by NMR (represented in dark blue color) and by X ray (represented in light blue color). The two black dots represent Z-scores of our model (-8.56) and template (-8.63). These scores indicate the overall quality of the modeled 3D structure of lanosterol 14-alpha demethylase of *C*. *albicans*. RMSD (0.35 Å) was calculated between the main chain atom of model and template, which indicated close homology. This ensured the reliability of the model.

#### Active Site Prediction

The best model thus obtained was submitted to DogSite scorer [23]. It predicted the existence of 10 different pockets. The one with highest P score was selected as the reliable active site pocket and considered to have a potential active site residue.

The 3D structure of ligands (compounds E1-E6 and FLC) were created by ChemDraw 8.0 and converted to the pdb file format after energy minimization (MOPAC). Ligand preparation was conducted by assigning Gastegier charges, merging nonpolar hydrogens, and saving it in pdbqt file format using AutoDock Tools (ADT) 4.2. The docking of the active molecules into the lanosterol 14α-demethylase (CYP51) modeled protein was done by positioning within the active site cavity. Molecular docking calculations were carried out with Autodock vina [24]. The conformation with the lowest binding free energy was used for analysis. All molecular docked models were prepared using PyMOL [25].

### RNA extraction and qRT-PCR

RNA extraction and gene expressions of *ERG11* gene in *C*. *albicans* ATCC90028, FLC- susceptible *C*. *albicans* 2281 and FLC- resistant *C*. *albicans* 3001 cells when exposed to MIC values of the test compounds E1-E6 or FLC were done as described previously [26]. Briefly, *C*. *albicans* ATCC90028, *C*. *albicans* 2281 and *C*. *albicans* 3001 were grown up to mid-log phase and were then exposed to MIC values of E1-E6 or FLC for 2 h in the same media. After incubation cells were washed and harvested by centrifuging at 3000g for 5 min. Pellets of approximately 1×10^6^ cells were homogenized and lysed with hydrochloric acid-treated glass beads (diameters, 0.5 mm; Braun Biotech, Melsungen, Germany) for 10 min, and total RNA was isolated using total GeneJET RNA kit (Fermentas Life Sciences, EU) following manufacturer’s instructions. Subsequently, the isolated RNA was digested with RNase free DNase I, and cDNA was synthesized using using RevertAid™ First Strand cDNA Synthesis Kit; K1622. The housekeeping gene ACT1 which was expressed to a similar extent under all conditions investigated was used as an endogenous control. Untreated cells and 1% DMSO represent the culture and negative controls, respectively.

Primer sets for *ACT1* and *Egr11* were respectively as follows: forward 5-TTTAAGAATTGATTTGGCT-3; reverse 5-GAAGATTGAGAAGAAGTTT-3 and forward 5-ATTGGTATTCTTATGGGTGGTCAACATAC-3; reverse 5- CCCAATACATCTATGTCTACCACCACC-3

The relative concentration of each transcript was determined using Real-Time 2xPCR ABI Power Sybergreen on Real Time PCR (ABI-7500). Each PCR protocol consisted of a primary denaturation step at 95°C for 20 s, followed by 30 cycles of denaturation at 95°C for 20 s, annealing at 45°C and extension at 72°C for 15 s. Each product was checked for its specificity in melting curve analyses and efficiency of the amplification was verified with standard curves for both the gene. Results were analysed by LightCycler Software 4.0. All qRT-PCR experiments were done in triplicate in two independent experiments for all the treated cell samples and results are shown as Mean ± standard deviation.

## Results and Discussion

### Synthesis

Eugenol, due to its antifungal properties was chosen as a reasonable scaffold for the present study and the hydroxyl group of the moiety on treatment with different sulfonyl chlorides under basic conditions resulted in the synthesis of the desired products as shown in Fig 1. Since the reaction of the -OH group of eugenol with p-toluenesulfonyl chloride is a tosylation reaction, all the synthesized derivatives were named after eugenol-tosylate. The structure of the synthesized compounds was established by elemental analyses, FTIR, ^1^H, ^13^C NMR and MS-ESI^+^-spectral studies.

FTIR spectra showed bands which significantly proved the formation of eugenol-tosylate and its congeners E1-E6. The absence of any free or H-bonded band at/or around 3200–3700 cm^-1^ corresponding to -OH and the presence of bands around 1143–1158 cm^-1^and 1308–1318 cm^-1^ corresponding to the sulfonyl group of the respective derivatives is an ample evidence. In the ^1^H and ^13^C NMR the absence of any signal for–OH proton and the appearance of characteristic peaks at expected chemical shifts and integral values for phenyl ring protons and methyl and ethyl protons in E2 and E6 provide significant indication for the formation of the derivatives E1-E6. The mass spectra of all the derivatives (E1-E6) showed [M+H]^+^ peak which is in accordance with the molecular formula of the compounds thus further confirming their formation. All the derivatives obeyed a similar pattern of fragmentation and in some derivatives the molecular ion peaks were observed as [M+Na^+^+H]^+^, [M+K^+^+H]^+^, [M+Na^+^] (Metal adduct ions). The data is presented in experimental section.

### Antifungal activity

The MICs of eugenol and its derivatives E1-E6 against all the tested clinical FLC- susceptible and FLC- resistant *Candida albicans* strains along with a reference strain of *C*. *albicans* ATCC 90028 are shown in Table 1. Evaluation of MIC values showed that eugenol has an MIC value of 500 μg/ml against all the tested *Candida* strains, however, its derivatives E1-E6 showed more potent *in vitro* activity with MIC values ranging from 1 to 62 μg/ml for clinical FLC- susceptible strains and 2 to 62 μg/ml for clinical FLC- resistant strains. The order of sensitivity on the basis of MIC values of the eugenol-tosylates is E5 > E3 > E4 > E1 > E2 > E6. The MICs of FLC against clinical FLC- susceptible *Candida* strains was 4–16 μg/ml and against clinical FLC- resistant *Candida* isolates was 125 μg/ml. These results also demonstrate that modification of eugenol decreases the MIC values drastically not only against FLC- susceptible *Candida* isolates but also against FLC- resistant isolates.

### Susceptibility in combination with fluconazole

The Fractional Inhibitory Concentration Indices (FICI) of E1-E6 combined with FLC were calculated to determine their possible interactions against all the tested *C*. *albicans* strains using the microbroth dilution method as previously described [18]. The data, shown in Table 3, for E1-E6 in combination with FLC in a 1:1 ratio, indicates synergistic, additive or indifferent interactions, while no antagonistic interaction was observed. Of the 150 combinations produced (Table 3), the FICI values against all the *Candida* strains ranged from 0.127 to 2.630. Out of 150 combinations tested, 36% were found to be synergistic while 41% and 23% were additive and indifferent, respectively (Table 3). Of all the combinations, the prominent synergistic interaction was observed between E5 and FLC against a resistant *C*. *albicans* 3081 (FICI = 0.127). Most of the synergistic interactions were observed between E5 and FLC (72%), while minimum synergistic interactions were observed between E4 and FLC (4%).

**Table 3:** The fractional inhibitory concentration index of E1-E6 tested in 1:1 combinations with fluconazole against different *C*. *albicans* strains.

Strains		E1 + FLC	INT	E2 + FLC	INT	E3+ FLC	INT	E4 + FLC	INT	E5 + FLC	INT	E6 + FLC	INT
**ATCC 90028**	∑FIC	0.630	ADD	0.750	ADD	0.255	**SYN**	1.130	IND	0.375	**SYN**	1.250	IND
**Clinical fluconazole susceptible strains**													
**074**	∑FIC	1.260	IND	0.380	**SYN**	0.193	**SYN**	0.564	ADD	0.625	ADD	0.630	ADD
**072 gr**	∑FIC	2.500	IND	0.531	ADD	0.750	ADD	1.268	IND	0.280	**SYN**	0.768	ADD
**072 big gr**	∑FIC	1.520	IND	0.760	ADD	0.625	ADD	1.268	IND	0.313	**SYN**	2.438	IND
**079 small gr**	∑FIC	1.520	IND	0.760	ADD	0.313	**SYN**	1.520	IND	0.625	ADD	1.520	IND
**002 B1**	∑FIC	2.000	IND	0.625	ADD	0.313	**SYN**	1.260	IND	0.281	**SYN**	0.750	ADD
**003 gr**	∑FIC	1.000	IND	0.255	**SYN**	0.625	ADD	0.630	ADD	0.313	**SYN**	0.750	ADD
**004 gr**	∑FIC	1.520	IND	1.000	IND	0.750	ADD	1.260	IND	0.625	ADD	0.630	ADD
**004 B1**	∑FIC	0.500	**SYN**	0.750	ADD	0.281	**SYN**	1.130	IND	0.146	**SYN**	0.630	ADD
**177**	∑FIC	0.375	**SYN**	0.375	**SYN**	0.193	**SYN**	2.520	IND	0.161	**SYN**	0.750	ADD
**2281**	∑FIC	0.630	ADD	0.750	ADD	1.000	IND	1.130	IND	0.500	**SYN**	1.260	IND
**2323**	∑FIC	0.750	ADD	1.000	IND	0.500	**SYN**	2.438	IND	0.375	**SYN**	0.630	ADD
**2367**	∑FIC	0.255	**SYN**	0.193	**SYN**	0.193	**SYN**	0.630	ADD	0.161	**SYN**	0.750	ADD
**2427**	∑FIC	0.500	**SYN**	0.750	ADD	0.161	**SYN**	1.500	IND	0.146	**SYN**	1.000	IND
**2434**	∑FIC	0.630	ADD	0.630	ADD	0.750	ADD	2.260	IND	0.193	**SYN**	2.260	IND
**2508**	∑FIC	1.000	IND	0.750	ADD	0.313	**SYN**	0.564	ADD	0.563	ADD	0.630	ADD
**Clinical fluconazole resistant strains**													
**3001**	∑FIC	0.322	**SYN**	0.564	ADD	0.564	ADD	0.748	ADD	0.266	**SYN**	0.374	ADD
**3048**	∑FIC	0.322	**SYN**	0.564	ADD	0.564	ADD	0.748	ADD	0.266	**SYN**	0.374	**SYN**
**3068**	∑FIC	0.282	**SYN**	0.532	ADD	0.258	**SYN**	0.374	**SYN**	0.516	ADD	0.624	ADD
**3087**	∑FIC	0.282	**SYN**	0.532	ADD	0.266	**SYN**	0.266	ADD	0.266	**SYN**	0.374	**SYN**
**3081**	∑FIC	0.282	**SYN**	0.532	ADD	0.129	**SYN**	1.284	IND	0.127	**SYN**	1.092	IND
**3140**	∑FIC	1.248	IND	0.282	**SYN**	0.282	**SYN**	0.748	ADD	0.532	ADD	0.748	ADD
**2357**	∑FIC	0.322	**SYN**	0.564	ADD	0.532	ADD	0.748	ADD	0.266	**SYN**	1.248	IND
**2261**	∑FIC	0.564	ADD	0.266	**SYN**	0.516	ADD	0.624	ADD	0.254	**SYN**	0.322	**SYN**
**3178**	∑FIC	0.532	ADD	0.266	**SYN**	0.258	**SYN**	0.748	ADD	1.016	IND	0.624	ADD

INT: Interpretation, ∑FIC is the sum of FIC_A_ and FIC_B_

**Fig 3:**
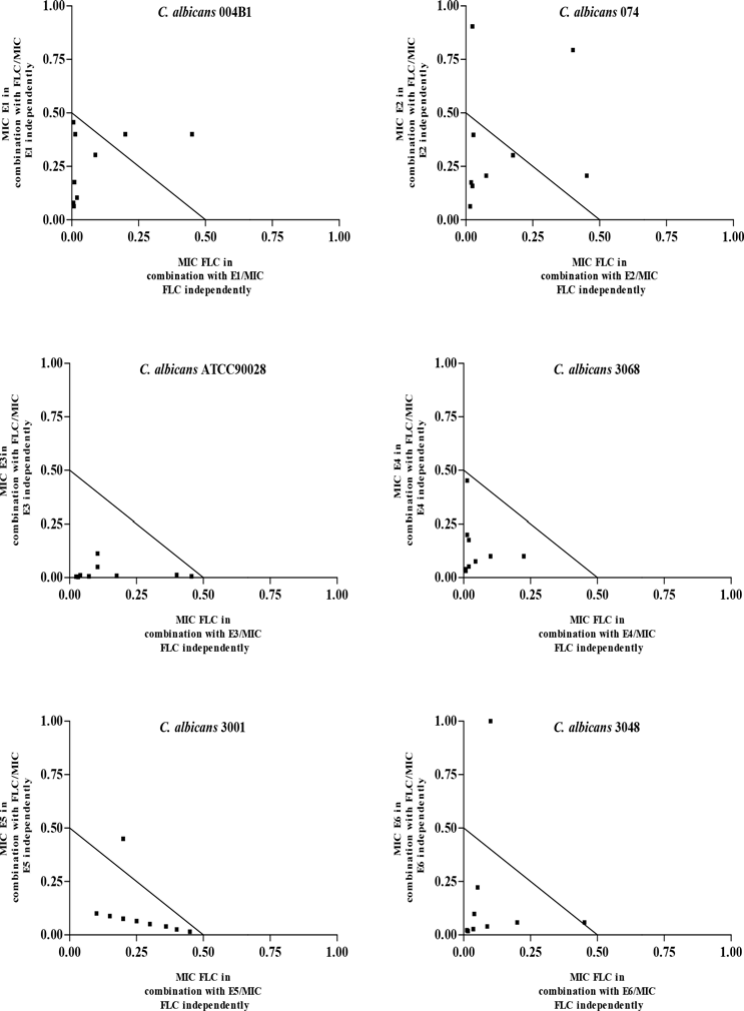
Isobolograms depicting synergistic interactions of eugenol-tosylates (E1 to E6) with fluconazole in nine different ratios against different *Candida albicans* isolates.

For 54 of the synergies in the 1:1 ratio, isobolograms were constructed, where nine different ratios of E1-E6 and FLC were blended and antimicrobial efficacies were determined. Representative isobolograms for each tosylate (E1-E6) are shown in Fig 3. From these results, marked synergistic interactions were observed with most of the combinations irrespective of the ratio, while all other ratios exhibited additive interactions. Interestingly, none of the combinations displayed indifferent or antagonistic effects. The combinations of E4 and FLC against *C*. *albicans* 3068 and E3 and FLC against *C*. *albicans* 90028 showed synergistic interactions for all the nine ratios examined. E5 and E6 in combination with FLC against *C*. *albicans* 3001 and *C*. *albicans* 3048, respectively, showed synergy for eight of the nine ratios assayed. These synergistic interactions are in agreement with the previous findings on synergistic combinations of antibiotics and newly synthesized compounds [27,28], and we also confirm the potential of these derivatives to chemosensitize the action of FLC and other azole drugs.

### Ergosterol Biosynthesis Assay and Docking Studies

**Fig 4:**
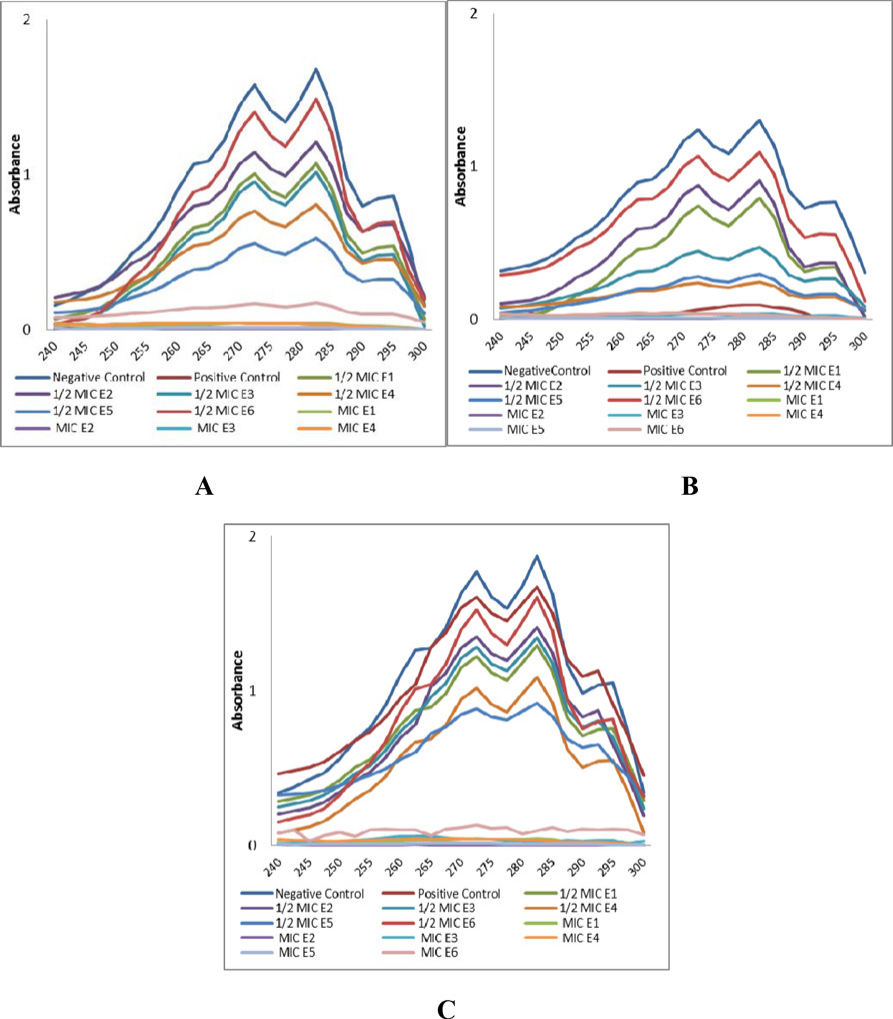
UV spectrophotometric sterol profiles of representative laboratory strain *C*. *albicans* ATCC 90028 (A), clinical fluconazole-susceptible *C*. *albicans* 2281 (B) and Clinical fluconazole-resistant *C*. *albicans* 3001 (C) strains after treatment with MIC and ½ MIC values of compounds E1 to E6. Isolates were grown for 16 h in sabouraud dextrose broth and sterols were extracted using alcoholic KOH and n-heptane from the treated and untreated cells and spectral profiles between 240 and 300 nm were determined. Control represents the culture cells without any treatment and positive control represents cells treated with 64 μg/ml of fluconazole.

**Fig 5:**
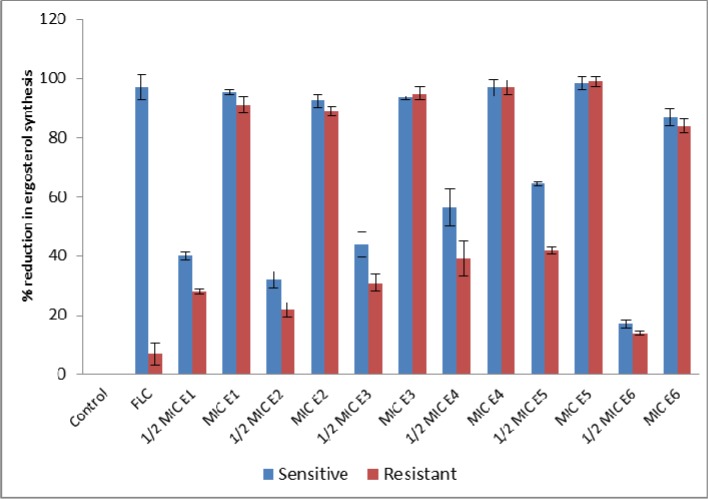
Ergosterol biosynthesis inhibition in different *Candida* strains upon treatment with compounds E1 to E6 at MIC and sub-MIC values. Ergosterol content was expressed by the percent reduction in the mean cellular ergosterol content compared with that of negative control cells grown without any treatment. FLC represents the positive control.

Sterol quantification was done in various *C*. *albicans* isolates following treatment with E1, E2, E3, E4, E5 and E6. The results obtained on ergosterol biosynthesis inhibition by test compounds in different *Candida* strains are represented in Fig 4 and the %-age reduction in ergosterol biosynthesis with respect to control cells is summarised in Fig 5. Complete inhibition of ergosterol biosynthesis was observed at MIC values of the test compounds, which are depicted by the flat lines, whereas at ½ MIC values of E1-E6, a dose dependent decrease in ergosterol biosynthesis was observed (Fig 4). Compared to the control cells (untreated), 18% to 64% ergosterol biosynthesis inhibition was noted when laboratory strain *C*. *albicans* ATCC90028 was treated with ½ MIC values of E1-E6, while this figure reached 89 to 100% when cells were treated with MIC values of E1-E6. For clinical fluconazole susceptible isolates ergosterol biosynthesis inhibition was observed to decrease in the range of 14% to 65% and 85% to 95% when cells were treated with ½ MIC and MIC values of E1-E6, respectively. Total cellular ergosterol content decrease was recorded as 14% to 42% and 84% to 99%, when clinical fluconazole resistant isolates were respectively treated with ½ MIC and MIC values of the test compounds E1-E6 (Fig 5). Fluconazole, as expected, inhibited 94% to 100% of ergosterol biosynthesis in the standard and clinical fluconazole susceptible strains of *Candida* whereas only 7% decrease was recorded for clinical fluconazole resistant strains. Ergosterol biosynthesis inhibition at sub-MIC values of the test compounds reached up to 65%, indicating that these *C*. *albicans* isolates belong to ergosterol tolerant class. Ergosterol tolerant class is a group of fungi which are tolerant to ergosterol biosynthesis inhibition and can grow in the deficiency of 14α- demethylation while intolerant class of fungi cannot survive in 14α- demethylation deficiency [20, 29]. In the latter group, MIC values of a drug are equal to the concentrations required to completely inhibit the ergosterol biosynthesis, whereas, for the tolerant class of fungi, MIC values are always higher than the concentrations required to inhibit the ergosterol biosynthesis.

**Fig 6:**
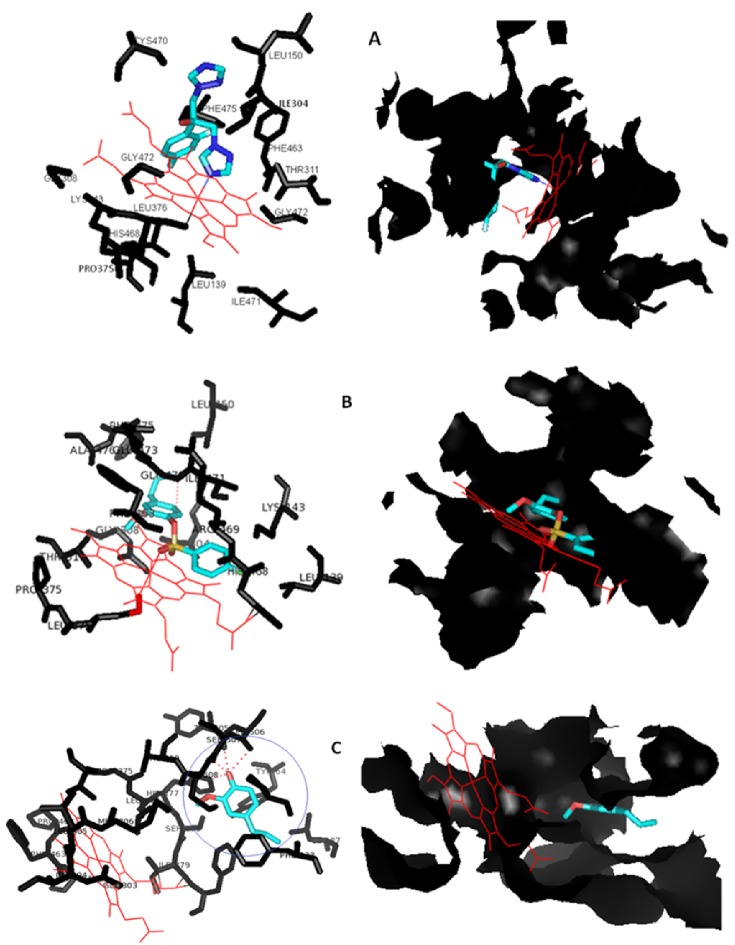
Docking and binding mode of (A) FLC (B) E5 and (C) EUG in the active site pocket of modelled CYP51 of *Candida albicans*. EUG showing interaction with other residues of the protein away from the pocket and heme.

To further validate the obtained results and to gain insight into the mechanism of action and binding interactions, all the compounds E1-E6 and reference azole drug FLC were docked into the active site of the modeled cytochrome P450 lanosterol 14α-demethylase of *C*. *albicans* using autodock 4.2 and autodock vina software package. The active site pocket consists of amino acid residues LEU139, LYS143, LEU150, ILE304, GLY308, THR311, PRO375, LEU376, PHE463, HIS468, ARG469, CYS470, ILE471, GLY472, GLU473, PHE475, ALA476 and Heme 360. Binding affinity values of the docked compounds E1-E6 were found to be in the range of -7.59 to -14.85 kcal mol^-1^ and that for FLC and EUG -16.5 kcal mol^-1^ and -9.54 kcal mol^-1^ respectively (Table 4). These results also suggest that FLC and the synthesized compounds interact with different residues of the protein and this interaction depends on the structure of the compounds. The presence of different substituents on the phenyl ring of the sulfonyl pendent also guides their interaction with the protein residues and heme. The docking result indicated that synthesized compounds were held in the active pocket by forming combination of hydrogen bonds, hydrophobic bonds and van der Waals interactions with the enzyme, besides showing interaction with the heme group. FLC coordinates with the heme iron by triazole nitrogen, besides showing other interactions within the pocket (Fig 6A). All the eugenol derivatives (E1-E6) showed binding in the active site of the enzyme, where the least binding energy and best results were displayed by E5 showing hydrogen bonding interaction with GLY472 residue of the protein (Fig 6B). Eugenol shows hydrogen-bonding interaction with HIS377, TYR505, SER506 and SER507 residues of the protein away from the heme (Fig 6C). The docking study values are given in Table 4. On the basis of activity data and docking results, it was found that all the compounds had the potential to inhibit 14α-demethylase of *C*. *albicans* and therefore can be considered as lead structures for further optimization.

**Table 4:** Results of docking study shown as binding affinity values and hydrogen bonding interactions of E1-E6 with aminoacid residues of cytochrome P450 lanosterol 14α-demethylase of *C*. *albicans*.

Compound	Binding affinity (kcal/mol)	H-bond interaction^a^	H-bond^b^	H-Bond length (Å)
**E1**	-9.72	THR311	1	3.2
**E2**	-8.49	GLY308	1	2.9
**E3**	-10.68	GLY472	1	2.6
**E4**	-10.54	THR311, ALA476	2	3.2
**E5**	-14.85	GLY472	1	3.2
**E6**	-7.59	ARG469, GLY472	2	3.2–3.3
**FLC**	-16.54	LEU204, THR311, PHE475, PHE463	4	2.6–3.3
**EUG**	-9.54	HIS377, TYR505, SER506, SER507	4	2.6–3.2

a = hydrogen bonds formed between the aminoacid residues of the modelled protein and the docked compounds.

b = Number of hydrogen bonds

From the above discussion it is clear that the incorporation of sulfonyl group in place of the hydroxyl group of eugenol in compounds E1-E6 enhances their activity; it could be because of the property of the sulfonyl group forming hydrogen bonding interactions with the active site residues of biological targets and also its incorporation into core ring structure with modified conformation allows snuggly fitting into the active site. The oxygen of the >S = O group of E5 also coordinates with the heme group of the modeled protein and therefore mimics fluconazole, in a similar way, when it coordinates with the heme group through its triazole nitrogen [4]. The presence of substituents on the phenyl ring of the sulfonyl pendent also has a marked effect on the biological profile of the molecule as seen in the present study. To define the chemical consequences of modifying the structure and explaining the biological results, it seems that the presence of electron releasing groups like CH_3_ and C_2_H_5_ in E2 and E6 decreases the activity and the presence of electron withdrawing groups in E3, E4 and E5, resulting in enhancement of activity compared to the unsubstituted E1. The presence of groups like Cl, NO_2_ and Br in E3, E4 and E5, however, resulted in disparity in activity, which could possibly be due the different nature of the groups. The chloro group resembles methyl in size, but differs electronically, considerably reducing ring Pka, compared to methyl or ethyl groups. Bromo is almost similar, but less electronegative compared to chloro, and nitro is considerably more electronegative and reduces ring pka drastically. Quite often, a change in the pKa has a strong effect on both the pharmacokinetic properties of the molecule and its binding affinity. For example, a strongly basic group may be required for binding within a certain lead series, but at the same time this basic group may also be found to result in compounds with low bioavailability due to the limited ability of a strong basic group to pass through membranes. The presence of different groups helps in finding an optimum between these conflicting effects. The effect of different substituents on physicochemical, biophysical, and pharmacological properties of a compound are also different. Also the substituents can interact directly with the protein, or indirectly by modulation of the polarity of other groups of the compound that interact with the protein. From the results of these preliminary SAR studies it can be inferred that optimum structural properties and balanced physicochemical properties are essential for a compound to show excellent activity. These results also suggest that the ergosterol biosynthesis inhibition is a fundamental mechanism of antifungal action of these test compounds (E1-E6), in a manner similar to that of fluconazole.

### Quantitative real-time PCR analysis

**Fig 7:**
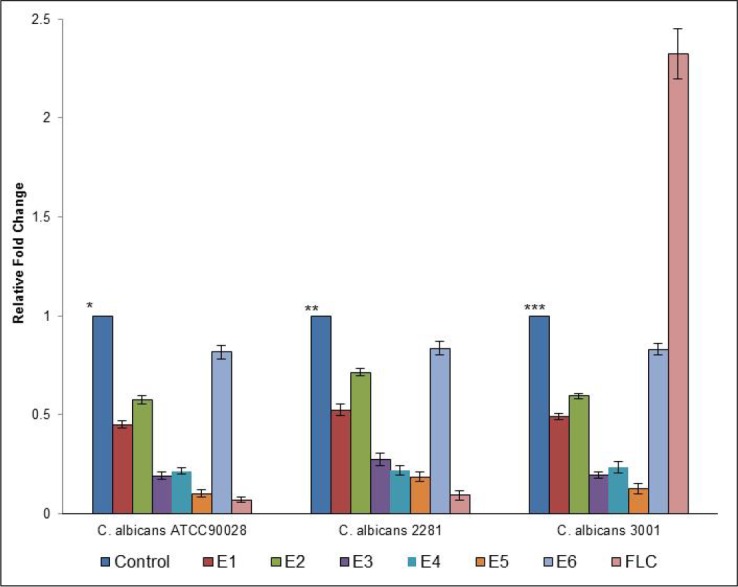
Relative expression of *ERG11* of *C*. *albicans* ATCC90028, *C*. *albicans* 2281 and *C*. *albicans* 3001 exposed to different eugenol-tosylates (E1-E6) or FLC at their respective MIC values. Control represent the compound free cells and had an average relative expression (of 3 independent recordings) with *3.124±0.124; **2.234±0.134; ***1.795±0.102

Since all the tested semi-synthetic derivatives E1-E6, inhibited ergosterol biosynthesis in different *Candida* isolates, we hypothesized changes in the corresponding gene expression levels in cells treated with E1-E6 or FLC. Studies have already reported the role of protein product, lanosterol 14α-demethylase, of *ERG 11* gene in ergosterol biosynthesis pathway [30,31] and the up regulation of this gene in azole resistance [31]. In view of this, one FLC- susceptible *C*. *albicans* 2281 and one FLC- resistant *C*. *albicans* 3001 along with *C*. *albicans* ATCC90028 were grown up to mid-log phase and were then exposed to MIC values of E1-E6 or FLC for 2 h in the same media. After incubation cells were harvested and RNA was extracted. From the extracted RNA, c-DNA was synthesized which was used as a template for a series of qRT-PCR experiments. The gene expression data observed by qRT-PCR is summarized in Fig 7. The expression of the housekeeping genes *ACT1* was also determined in every set of experiments. The expression of *ERG11* gene is shown as relative values in comparison to the control (untreated) that were set to one. From the results, down regulation of the *ERG11* gene in different *C*. *albicans* isolates to varying extents were observed when cells were exposed to these derivatives. As expected, FLC also down regulated the *ERG11* gene in *C*. *albicans* ATCC90028 and *C*. *albicans* 2281, while up regulation of *ERG11* was observed when FLC- resistant *C*. *albicans* 3001 cells were exposed to the FLC. The up regulation of *ERG11* gene in resistant cells following the exposure of FLC was congruent with the results observed previously and has been shown to be induced by the transcription factor encoded by the *UPC2* gene [32]. Up regulation of *ERG11* resulting in the increased production of ERG11p are often associated with the azole, polyene and azole-polyene cross-resistances [33]. The variation in the expression of this gene in the presence of FLC in different FLC- susceptible and FLC- resistant isolates in the present study also confirms the role of this gene in azole resistance. Our results with gene expression agree with observed changes in the inhibition of ergosterol biosynthesis as observed in sterol quantification assay. In this study, we only focused on the *ERG11* gene as a specific regulator for ergosterol biosynthesis in *C*. *albicans*; however, several other genes are also involved in this process. It is also believed that most of these specific gene regulations involve similar promoter structure [34,35], suggesting the possibility of these semi-synthetic derivatives for influenced regulation at their loci as well. Although resistance against available antifungals is very common, it has been extensively studied that eugenol showed antifungal activities with several different targets in fungal cells [15,36,37] and therefore the chance of resistance development against these compounds is very minimal [37].

The *in vitro* hemolytic assay is a possible screening tool to gauge *in vivo* toxicity to host cells [15]. With this assay, eugenol has already been reported to possess least or no cytotoxicity against the human blood cells as compared to the known antifungals (amphotericin B and fluconazole). It has also been reported that concentration of eugenol when used in combination with other drugs is far below its cytotoxic values [38]. However, these observations do not warrant that the congeners do not have any cytotoxicity and therefore further cytotoxicity and *in vivo* studies are required to delineate the cytotoxic effects of these compounds.

In conclusion, modification of eugenol structure into its semi-synthetic analogues and combination with fluconazole is a promising strategy to treat azole resistant *Candida* strains on account of their significant antifungal activity against different FLC- susceptible and FLC- resistant strains by inhibiting ergosterol biosynthesis and also down regulating its related gene at MIC values. The structure, however, further needs to be optimized by substituting the phenyl ring of the sulfonyl pendent with electronically different groups and further studies are needed to determine the underlying mechanisms of the antifungal enhancements and the synergistic interactions. In addition the potential of combination therapy use *in vivo* warrants investigation.

## Supporting Information

S1 Fig Flow Chart diagram for homology modelling (TIF)

S2 Fig Ramachandran plot of Modelled structure (TIF)

S3 Fig Ramachandran plot of 4KOF (TIF)

S4 Fig Plot of Z-score of 4K0F (A) and modeled structure (B) (TIF)
