# Supplementary figures and images for: Synergistic Interactions of Eugenol-tosylate and Its Congeners with Fluconazole against Candida albicans
Source: PLoS One. 2015 Dec 22;10(12):e0145053. doi: 10.1371/journal.pone.0145053 (PMC4980062; doi:10.1371/journal.pone.0145053)

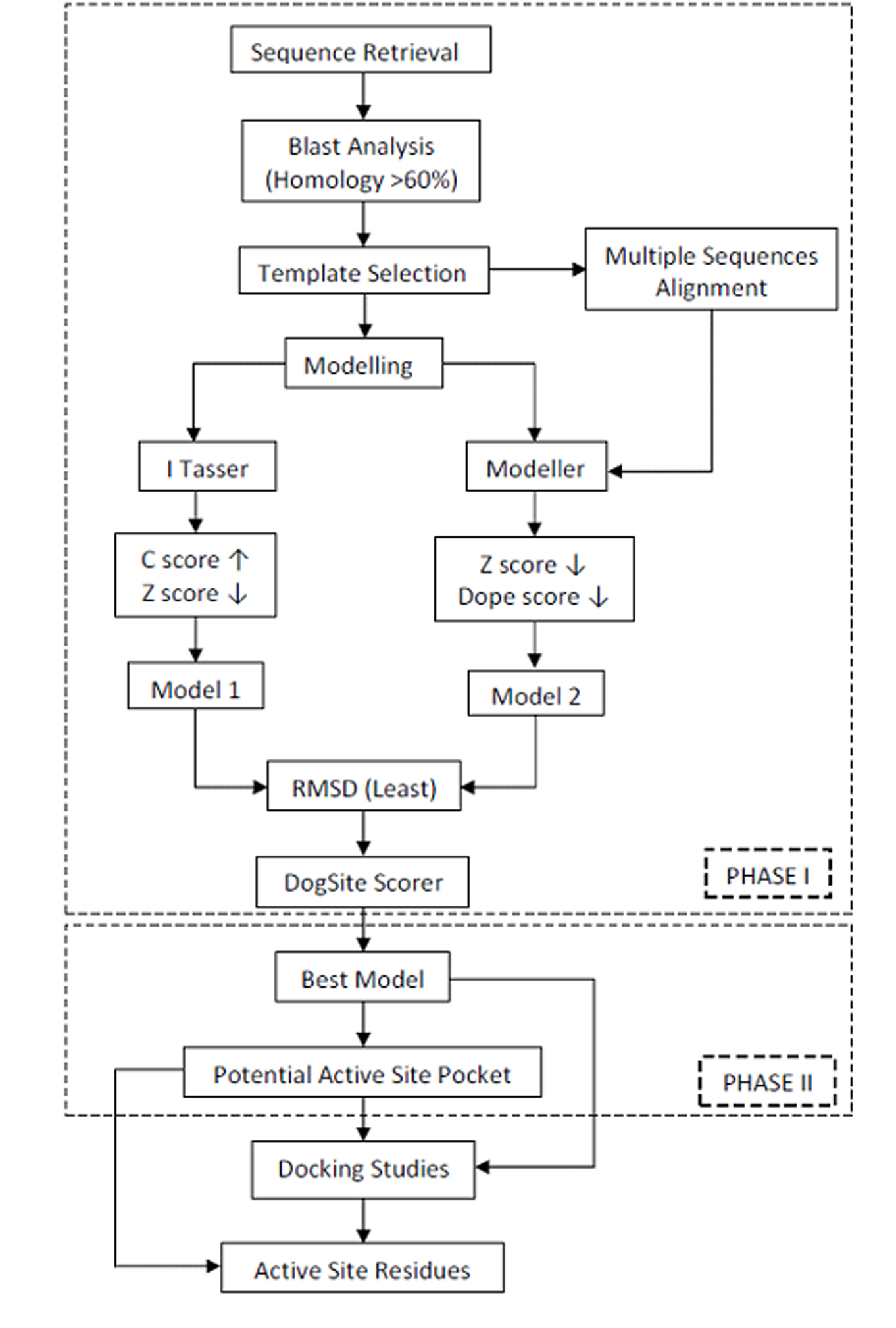

Supplement: S1 Fig — (TIF) [file pone.0145053.s001.tif]

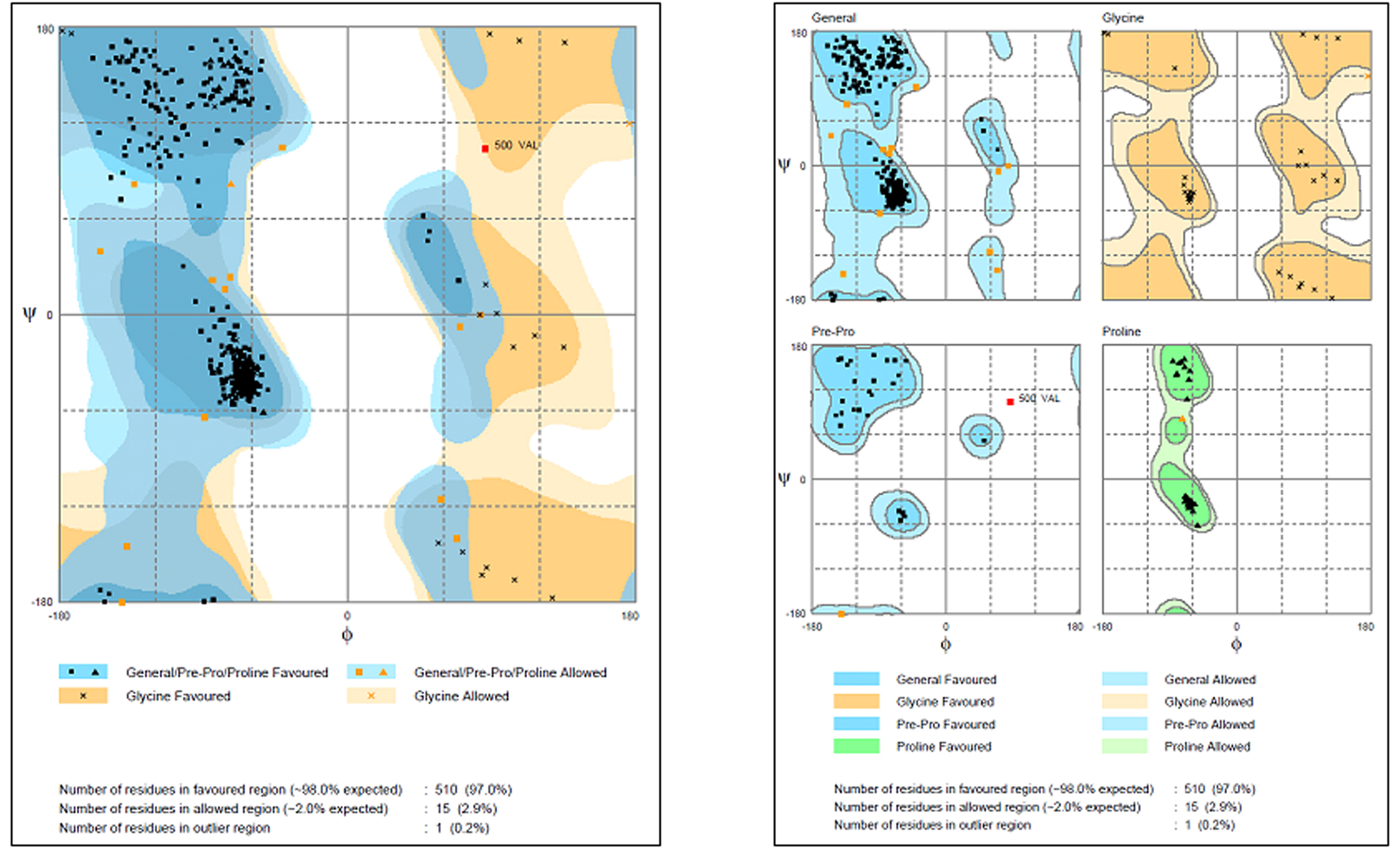

Supplement: S2 Fig — (TIF) [file pone.0145053.s002.tif]

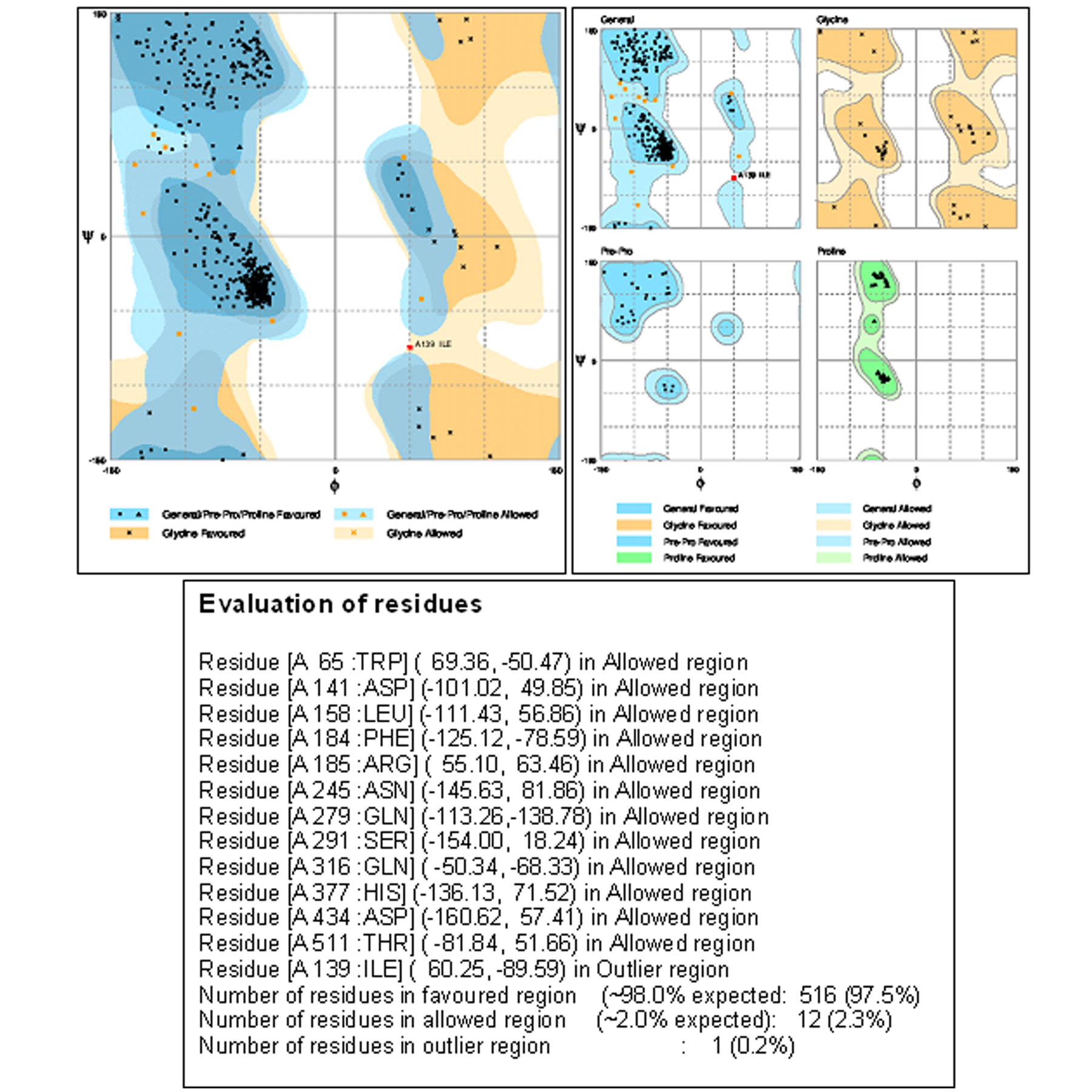

Supplement: S3 Fig — (TIF) [file pone.0145053.s003.tif]

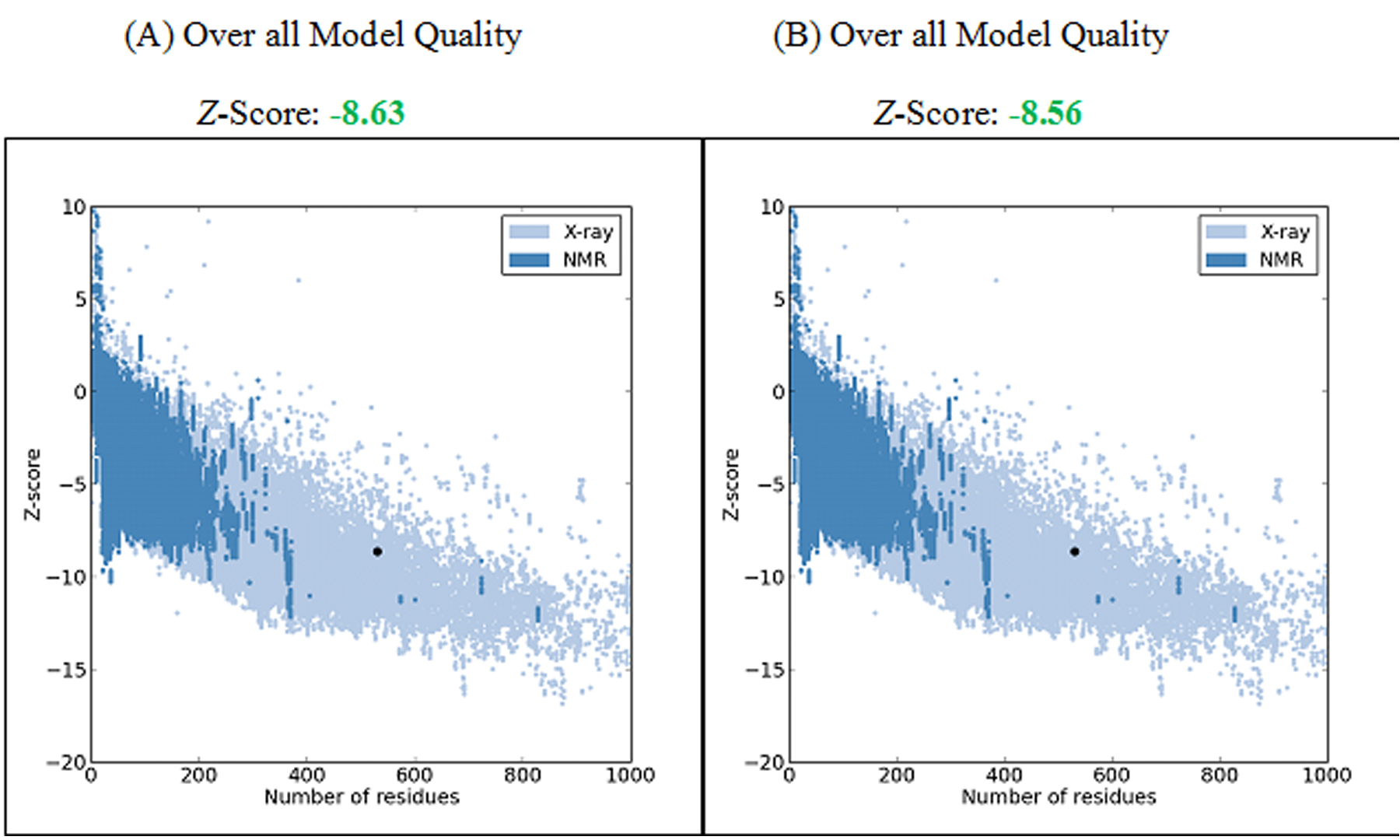

Supplement: S4 Fig — (TIF) [file pone.0145053.s004.tif]
